# Integrating Dimensional Personality and Autistic Traits to Predict Suicidal Ideation, Suicide Attempts, and Nonsuicidal Self‐Injury in Autistic Adults

**DOI:** 10.1002/aur.70202

**Published:** 2026-02-17

**Authors:** Aliona Tsypes, Timothy A. Allen, Ligia Antezana, Kelly B. Beck, Caitlin M. Conner, Lori N. Scott, Carla A. Mazefsky

**Affiliations:** ^1^ Department of Psychiatry University of Pittsburgh School of Medicine Pittsburgh Pennsylvania USA

**Keywords:** adults, autism, CATI, NSSI, personality, PID‐5, suicide

## Abstract

Given the elevated rates of suicidal ideation, suicide attempts, and nonsuicidal self‐injury (NSSI) in autistic adults, we examined whether autism‐informed traits and transdiagnostic personality tendencies jointly relate to these outcomes. One hundred and two adults with clinician‐diagnosed autism completed structured clinical interview assessments of lifetime histories of suicidal ideation, attempts, and NSSI. Predictors were six Comprehensive Autistic Trait Inventory (CATI) subscales and selected Personality Inventory for DSM‐5 Short Form (PID‐5‐SF) domains and facets. We fit CATI‐only, PID‐5 domain, and facet models, then combined significant predictors and refit with age, sex, and IQ as covariates. Shared variance between PID‐5‐SF facet Anhedonia and CATI Social Interactions showed suppression in joint models, and latent variable modeling confirmed that their shared variance—indexing overlapping reward and social disengagement—was the most consistent correlate of risk across outcomes. PID‐5‐SF facet Emotional Lability was robustly related to NSSI and to ideation severity. CATI Self‐Regulatory Behaviors predicted NSSI. PID‐5‐SF domain Disinhibition showed no associations. Higher IQ showed a modest protective effect for attempts. Findings highlight central roles of reward‐related processes and affective volatility, with added contributions from interpersonal strain and self‐regulation. Combining CATI with PID‐5 yields complementary targets for assessment and intervention. Key strengths include a clinician‐diagnosed autistic sample, a rare direct comparison of people with lifetime suicidal ideation vs. suicide attempts, and an integrated trait framework that moves the field beyond prevalence toward trait‐informed risk. Findings support brief screening for anhedonia and emotional lability, autism‐adapted behavioral activation, rapid arousal‐reduction skills, and attention to social communication needs that may impede disclosure and help‐seeking.

## Lay Summary

In 102 clinician‐diagnosed autistic adults, lower enjoyment of daily activities and greater social difficulties were linked to higher risk of suicidal thoughts, suicide attempts, and self‐harm without intent to die. Rapid mood swings also related to self‐harm and more severe suicidal thinking. These results support brief screening for low pleasure and mood swings, efforts to reduce social strain, and the use of autism‐adapted behavioral activation and quick calming skills.

## 1 Introduction

Autistic adults experience significantly elevated rates of suicidal ideation, suicide attempts, and nonsuicidal self‐injury (NSSI), with up to one‐third having attempted suicide (van Bentum et al. 2024; Brown et al. 2024; Cassidy et al. 2018; Maddox et al. 2017; Newell et al. 2023; Schwartzman et al. 2025; Steenfeldt‐Kristensen et al. 2020). Although self‐injurious thoughts and behaviors (SITB) are intercorrelated, they are distinct processes with different functions. Specifically, NSSI is separate from suicidal thinking and behavior due to its lack of suicidal intent (Hooley et al. 2020; Klonsky et al. 2014; Nock and Favazza 2009). Further, contemporary ideation‐to‐action theories (Klonsky and May 2015; O'Connor and Kirtley 2018; Van Orden et al. 2010) separate the formation of suicidal ideation from a transition to suicide attempts. While the scale of this issue is now well‐documented, the field has only begun to move beyond establishing prevalence toward identifying the individual difference factors that differentiate autistic people who engage in NSSI, those who contemplate suicide, and those who attempt it.

Personality traits, typically conceptualized as relatively stable patterns of thinking, feeling, and behaving, provide a promising lens for understanding these distinct thoughts and behaviors. These traits describe how intensely and how often people experience certain emotional states and how readily they translate urges into action. Such a lens is particularly relevant for autism, where chronic stressors (e.g., sensory overload, social isolation, or exclusion) are pervasive and can overtax coping resources (Beck et al. 2024; Lai 2023; Mournet et al. 2024; Zhuang et al. 2023). When such pressures intersect with a tendency toward strong emotional reactivity or impulsive responding, crisis risk may escalate; conversely, personality traits linked to planning or emotional stability may buffer against SITB. Despite this logic, empirical work on personality trait‐based vulnerability to SITB in autistic adults remains sparse, commonly limited to measures of depression or general social support (Brown et al. 2024; Mournet et al. 2024).

An autism‐informed perspective is a useful starting point for this investigation. The Comprehensive Autistic Trait Inventory (CATI; English et al. 2021, 2025) offers a modern, community‐informed assessment of six domains that are central to autistic experience: Social Interactions, Communication, Social Camouflage, Self‐Regulatory (Repetitive) Behaviors, Cognitive Flexibility, and Sensory Sensitivity. The CATI was designed to capture diagnostically relevant autistic traits, and its neutral, community‐vetted wording makes it a less stigmatizing option than many legacy instruments. CATI improves on legacy instruments by the inclusion of domains (i.e., camouflaging) that capture autistic adult experiences beyond just diagnostic features. In doing so, the CATI also measures features that have clear relevance to SITB (Brown et al. 2024; Cassidy et al. 2018). Social Camouflage indexes masking and effortful compensatory strategies that autistic people use to appear non‐autistic. Self‐Regulatory (Repetitive) Behaviors describe repetitive or patterned actions that can help alleviate stress or anxiety. Cognitive Flexibility taps preference for sameness and difficulty with change, which can intensify distress during disruptions. Sensory Sensitivity indexes reactivity to environmental stimuli that can precipitate overload. The Social Interactions and Communication scales capture the desire for social contact, perceived competence in interactions, and use and understanding of nonverbal communication. By measuring these specific domains, the CATI allows for a direct examination of how core autistic experiences contribute to SITB. However, despite the strengths of CATI in overcoming numerous limitations of prior self‐report measures of autistic traits (English et al. 2021, 2025), no studies of which we are aware have examined the links between CATI‐assessed traits and SITB in autistic adults, which was our first goal.

Broader trait taxonomies may also have utility in understanding SITB within autism, as they describe general personality tendencies that cut across diagnostic groups and show partial (Widjaja et al. 2025) or full (Wakabayashi et al. 2006) independence from autistic traits. Considering these personality traits may provide additional insights into vulnerabilities for SITB that have not been previously examined in autistic samples. Factor‐analytic research has consistently shown that both adaptive and maladaptive features of personality can be described by a single structural framework that includes five broad domains: Neuroticism, Extraversion, Agreeableness, Conscientiousness, and Openness (John et al. 2008; Markon et al. 2005). The DSM‐5 dimensional model^1^ of personality similarly groups related tendencies into five domains: Negative Affectivity, Detachment, Antagonism, Disinhibition, and Psychoticism. These domains roughly parallel the Big Five (though the mapping from Openness to Psychoticism is not one‐to‐one; see Suzuki et al. 2017), while emphasizing the tendencies that can create persistent strain in daily life. Negative Affectivity reflects the propensity for intense negative emotion and volatility, Detachment reflects reduced interest in social engagement and diminished pleasure, Antagonism reflects interpersonal exploitation and self‐focus, Disinhibition reflects impulsivity and limited behavioral regulation, and Psychoticism reflects unconventional thought and perception. The Personality Inventory for DSM‐5 (PID‐5; Krueger et al. 2012) operationalizes these domains and, crucially for the present study, provides facet‐level indicators that enable a fine‐grained view of the specific tendencies that may drive risk. In the current study, we focus on the personality domains of Negative Affectivity, Detachment, and Disinhibition, and their facets, as these traits have the strongest theoretical and empirical ties to suicidal ideation, attempts, and NSSI.

Internalizing and externalizing tendencies appear to support different stages of the suicidal process, although evidence largely comes from non‐autistic samples. Within the motivational–volitional framework, internalizing processes contribute to the onset and maintenance of suicidal ideation, whereas externalizing processes help drive the transition from thoughts to suicidal behavior (O'Connor and Kirtley 2018). Consistent with this, impulsivity (a facet of Disinhibition) has differentiated people with histories of suicidal ideation from those with attempts in several studies (e.g., Dhingra et al. 2015; Nock et al. 2018). Related work shows that higher Negative Affectivity can amplify the link between interpersonal stress and suicidal ideation, while Disinhibition is more closely tied to attempts (Allen et al. 2022). With regard to NSSI, meta‐analytic and longitudinal studies indicate associations with both high Negative Affectivity (especially emotional lability; Peters et al. 2016) and impulsivity (e.g., Brezo et al. 2006; Hamza et al. 2015; You et al. 2016). Further, empirical work robustly links SITB with anhedonia, a construct captured by the Anhedonia facet of Detachment, and with broader reward‐related (e.g., Antezana et al. 2024; Bettis et al. 2022; Ducasse et al. 2018; Tsypes et al. 2018, 2019, 2021) and interpersonal processes (Hutchinson et al. 2025; Janssens et al. 2024). Taken together, consideration of transdiagnostic personality features has contributed to an improved understanding of SITB in non‐autistic samples, differentially implicating Negative Affectivity, Detachment, and Disinhibition—and in some cases, individual facets within these domains—in suicidal ideation, suicide attempts, and NSSI. The second goal of the present study was to examine how transdiagnostic personality traits, viewed through the lens of the Big Five framework, contribute to SITB in autistic adults.

Autism shows a distinctive trait profile that further motivates *combining* both autism‐informed and broader trait taxonomy‐informed measures. Meta‐analytic work indicates lower average levels across Big Five domains compared to non‐autistic peers (Lodi‐Smith et al. 2019). Conceptual accounts also suggest that autism is characterized by reduced draw to novelty and social reward (Rogers et al. 2023), which may relate to SITB in autistic people (Hedley et al. 2021; Reid et al. 2024). Thus, considering autistic and general personality traits in conjunction may offer a more nuanced picture of shared and unique risk factors for SITB. CATI targets experiences central to autistic life that are not fully represented in broad trait models, whereas the PID‐5 provides a broader, well‐validated dimensional taxonomy of personality that may help to identify features that are underassessed in autistic people but that are nonetheless critical to the development of SITB. Empirical work also suggests partial overlap. For example, social autism traits map onto Extraversion/Detachment (Widjaja et al. 2025), but cognitive and behavioral features are less well represented (Michelini et al. 2024; Stanton et al. 2021; Widjaja et al. 2025). Together, these points underscore the utility of combining CATI and PID‐5 to get a full picture of risk for SITB in autistic adults, which was the third goal of the present study.

In sum, the present study sought to integrate CATI and PID‐5 to examine trait contributions to SITB among autistic adults capable of consenting and self‐reporting. Specifically, we focused on lifetime histories of NSSI and suicide attempts, as well as suicidal ideation severity (the highest stage ever reached on the ideation‐to‐plan trajectory) and suicidal ideation intensity (strength and persistence during the worst lifetime episode). We further tested whether trait profiles distinguished people with lifetime suicidal ideation from those with a history of at least one suicide attempt (ideation‐to‐attempt transition). We focused on three questions: (1) Are CATI domains associated with SITB? (2) Are PID‐5 domains and facets associated with SITB? (3) Of the CATI domains and PID‐5 facets identified as significant in steps (1) and (2), which remain the strongest predictors when considered together? Guided by ideation‐to‐action theories (Klonsky and May 2015; O'Connor and Kirtley 2018; Van Orden et al. 2010), we hypothesized that Negative Affectivity and Detachment (particularly the Anhedonia facet) would relate most strongly to ideation, Disinhibition would distinguish people with ideation versus attempt history, and NSSI would show contributions from all three domains (particularly Negative Affectivity domain and its facet Emotional Lability). Analyses of CATI domains were exploratory.

## 2 Methods

### 2.1 Participants

Participants were recruited from community partners, local sources, and research registries. Recruitment approaches are detailed further in Mazefsky et al. (2025). Participants in this study included 102 autistic adults (67 female) between the ages of 18 and 65 (mean = 31.06, SD = 9.27) with self‐reported ability to read English. In this sample, 41 participants had lifetime suicidal ideation only, 38 had at least one suicide attempt, and 63 reported lifetime NSSI engagement. Autism diagnosis was determined through the Autism Diagnostic Observation Schedule–Second Edition (ADOS‐2; Lord et al. 2012) administered by a trained clinician and a brief clinical interview based on the DSM‐5 checklist (Williams and Lewis 2021). Participants were excluded if a clinician, tester, or investigator determined that they would be unable to complete the study tasks required of the parent study (see Mazefsky et al. 2025). Additional exclusion criteria included the presence of current mania, psychosis, or severe alcohol/substance use that could interfere with participation. Written informed consent was obtained from all participants prior to study participation, and the Institutional Review Board approved all procedures. Mean IQ was 115.70 (SD = 13.97); 41 participants held a bachelor's degree or higher; 47 had some college, associate, or vocational/technical training; 12 had a high‐school diploma or equivalent; 2 did not report their education.

### 2.2 Measures

#### 2.2.1 Columbia‐Suicide Severity Rating Scale (C‐SSRS; Posner et al. 2011)

The C‐SSRS is a semi‐structured clinical interview designed to assess lifetime SITB. Core questions were dichotomous (ideation, attempts, preparatory acts), followed by probes on ideation and attempt characteristics. Administration was adapted for autistic adults: a five‐step “thermometer” to anchor ideation from passive wish to plan with intent, calendar grids to date attempts, and written versions of longer questions and response options. Interviewers practiced under supervision until their ratings matched study standards and met weekly to settle scoring questions. We analyzed the highest lifetime ideation severity (highest score: intent with specific plan) and worst‐episode ideation intensity among ideators (sum of frequency, duration, reasons, deterrents, controllability).

#### 2.2.2 Self‐Injurious Thoughts and Behaviors Interview (SITBI; Nock et al. 2007)

Selected items captured NSSI history and characteristics. We incorporated visuals and an autism‐focused NSSI framework (Antezana et al. 2025).

#### 2.2.3 Wechsler Abbreviated Scale of Intelligence, Second Edition (WASI‐II; Wechsler 2011)

The WASI‐II provides a general estimate of cognitive ability and is normed for ages 6–90 years. For this study, an examiner administered the two‐subscale version, which consisted of one verbal and one perceptual reasoning subtest, yielding an overall IQ score. The 2‐subscale IQ demonstrates strong validity and reliability with a full‐scale IQ score (Wechsler 2011).

#### 2.2.4 Personality Inventory for DSM‐5 Short Form (PID‐5‐SF; Maples et al. 2015)

The PID‐5‐SF is a 100‐item self‐report measure of personality traits. Items are rated from 0 (“very false or often false”) to 3 (“very true or often true”), and higher scores indicate greater expression of each trait. Negative Affectivity (*α* = 0.85) facets are Emotional Lability (*α* = 0.87), Anxiousness (*α* = 0.87), and Separation Insecurity (*α* = 0.80). Detachment (*α* = 0.84) facets are Withdrawal (*α* = 0.80), Anhedonia (*α* = 0.87), and Intimacy Avoidance (*α* = 0.87). Disinhibition (*α* = 0.82) facets are Impulsivity (*α* = 0.87), Distractibility (*α* = 0.80), and Irresponsibility (α = 0.57).

#### 2.2.5 Comprehensive Autistic Trait Inventory (CATI; English et al. 2021, 2025)

The CATI is a 42‐item self‐report measure of autistic traits. Respondents rate each item from 1 (“strongly disagree”) to 5 (“strongly agree”). Items form six subscales: Social Interactions (α = 0.86), Communication (α = 0.69), Social Camouflage (α = 0.74), Self‐Regulatory (Repetitive) Behaviors (α = 0.74), Cognitive Rigidity/Flexibility (α = 0.74), and Sensory Sensitivity (α = 0.69), with higher scores reflecting greater endorsement of autistic traits. *α* = 0.74 for Self‐Regulatory Behaviors, 0.74 for Cognitive Rigidity/Flexibility, 0.74 for Social Camouflage, 0.69 for Sensory Sensitivity, 0.86 for Social Interactions, and 0.69 for Communication.

## 3 Results

### 3.1 Analytical Approach

For each outcome (NSSI, suicide attempts, suicidal ideation intensity, suicidal ideation severity, and ideation‐to‐attempt transition), we fit three models in sequence: (1) a six‐subscale CATI model; (2) a three‐domain PID‐5 model (Negative Affectivity, Disinhibition, Detachment); and (3) three‐facet PID‐5 models within each PID‐5 domain. For a domain, if none of its three facets were significant, we advanced the domain score (if significant) to the joint model. Given the nested structure of facets within a given domain, if any facet was significant, we advanced only the significant facet(s) and omitted the domain score. The final joint model included all significant CATI subscales plus these PID‐5 predictors and was re‐estimated with covariates (age, sex,^2^ IQ). All continuous predictors (CATI subscales, PID‐5 facets, IQ, and age) were standardized (*z*‐scored) prior to analysis. Higher CATI and PID‐5 scores indicate greater difficulty in the corresponding domain or facet. Table 1 presents descriptive statistics for sample characteristics, SITB outcomes, and predictor variables, including means, standard deviations, and observed ranges for continuous measures, and frequencies and percentages for binary outcomes.

**TABLE 1:** Descriptive statistics for sample characteristics, SITB outcomes, and predictor variables (*N* = 102).

Variable	*n*	*M* (SD) or *n* (%)	Range
Demographics			
Age (years)	101	31.06 (9.27)	18–55.09
Sex at birth (female)	101	67 (66.3%)	0, 1
IQ (WASI‐II Full Scale)	101	115.7 (13.97)	64–148
SITB outcomes: binary (presence/absence)			
Lifetime NSSI (yes/no)	102	63 (61.8%)	0, 1
Lifetime suicide attempt (yes/no)	102	38 (37.3%)	0, 1
Lifetime suicidal ideation only (no attempt)	102	41 (40.2%)	0, 1
NSSI methods (among those with lifetime NSSI)			
Cut or carved skin	63	44 (69.8%)	—
Hit self (resulting in bruising)	63	35 (55.6%)	—
Picked at a wound	63	32 (50.8%)	—
Picked/scraped/erased skin (drawing blood)	63	32 (50.8%)	—
Pulled hair out	63	19 (30.2%)	—
Bit self (drawing blood)	63	17 (27%)	—
Burned skin	63	16 (25.4%)	—
Inserted objects under nails/skin	63	14 (22.2%)	—
Gave self a tattoo	63	5 (7.9%)	—
Other	63	23 (36.5%)	—
Methods per participant	63	3.8 (2.2)	1–10
SITB outcomes: continuous			
SI intensity (ideators only)	90	16.43 (4.37)	4–25
SI severity (lifetime)	102	3.07 (1.66)	0–5
CATI subscales			
Self‐Regulatory Behaviors	102	29.7 (4.65)	11–35
Cognitive Rigidity	102	29.52 (4.44)	15–35
Social Camouflaging	102	28.5 (4.62)	14–35
Sensory Sensitivity	102	29.1 (4.39)	13–35
Social Interactions	102	27.3 (5.4)	7–35
Communication	102	22.12 (4.59)	7–31
PID‐5‐SF facets: negative affectivity (mean scores)			
Emotional Lability	102	1.29 (0.84)	0–3
Anxiousness	102	1.8 (0.87)	0–3
Separation Insecurity	102	1.2 (0.89)	0–3
PID‐5‐SF facets: detachment (mean scores)			
Anhedonia	102	1.16 (0.83)	0–2.75
Withdrawal	102	1.41 (0.72)	0–3
Intimacy Avoidance	102	0.95 (0.89)	0–3
PID‐5‐SF facets: disinhibition (mean scores)			
Impulsivity	102	0.91 (0.83)	0–2.75
Distractibility	102	2.06 (0.71)	0–3
Irresponsibility	102	0.55 (0.53)	0–2

*Note:* Binary outcomes are coded as presence (1) vs. absence (0) based on the Columbia‐Suicide Severity Rating Scale (C‐SSRS) and Self‐Injurious Thoughts and Behaviors Interview (SITBI) lifetime endorsement. SI intensity is calculated as the sum of five C‐SSRS intensity items (frequency, duration, reasons, deterrents, controllability; each item scored 0–5). SI severity reflects the highest lifetime ideation severity on the C‐SSRS (0–5; highest score: intent with specific plan). NSSI methods were assessed using the SITBI. Higher CATI and PID‐5 scores indicate greater difficulty within the corresponding subscale or facet.

Abbreviations: CATI = Comprehensive Autistic Trait Inventory; NSSI = nonsuicidal self‐injury; PID‐5‐SF = Personality Inventory for DSM‐5 Short Form; SI = suicidal ideation; SITB = self‐injurious thoughts and behaviors; WASI‐II = Wechsler Abbreviated Scale of Intelligence, Second Edition.

#### 3.1.1 Lifetime NSSI (Table 2)

*CATI six‐subscale model*: Self‐Regulatory (Repetitive) Behaviors and Social Interactions were positively associated with NSSI; Communication was negatively associated with NSSI.

**TABLE 2:** Predictors of lifetime NSSI in 102 autistic adults: CATI screens, PID‐5 screens, and joint logistic models with standardized predictors.

Model	Predictor	Estimate	SE	*z*	*p*
CATI six‐subscale	**Self‐Regulatory (Repetitive) Behaviors**	**1.27**	**0.56**	**2.26**	**0.024**
	Cognitive Flexibility	0.51	0.37	1.39	0.164
	Social Camouflage	−0.51	0.43	−1.19	0.236
	Sensory Sensitivity	0.52	0.43	1.23	0.219
	**Social Interactions**	**0.95**	**0.38**	**2.52**	**0.012**
	**Communication**	**−0.82**	**0.40**	**−2.05**	**0.041**
PID‐5 domains	Disinhibition	0.04	0.28	0.15	0.882
	**Detachment**	**0.53**	**0.25**	**2.11**	**0.035**
	Negative Affectivity	0.48	0.27	1.76	0.079
PID‐5 facets	**Emotional Lability**	**1.34**	**0.35**	**3.84**	**< 0.001**
	Anxiousness	−0.38	0.31	−1.22	0.223
	Separation Insecurity	−0.13	0.28	−0.47	0.641
	Withdrawal	−0.04	0.31	−0.13	0.900
	Anhedonia	0.60	0.32	1.88	0.060
	Intimacy Avoidance	0.19	0.27	0.72	0.472
	Irresponsibility	−0.31	0.31	−1.00	0.320
	Impulsivity	−0.01	0.35	−0.03	0.973
	Distractibility	0.02	0.32	0.08	0.940
Final (unadjusted)	**Self‐Regulatory (Repetitive) Behaviors**	**1.53**	**0.59**	**2.60**	**0.009**
	Social Interactions	1.52	0.43	1.22	0.223
	**Communication**	**−0.93**	**0.42**	**−2.22**	**0.026**
	**Emotional Lability**	**0.99**	**0.29**	**3.44**	**< 0.001**
	Detachment	0.41	0.32	1.25	0.210
Final (adjusted for age, sex, IQ)	**Self‐Regulatory (Repetitive) Behaviors**	**1.67**	**0.67**	**2.52**	**0.012**
	Social Interactions	0.82	0.48	1.72	0.086
	**Communication**	**−0.86**	**0.43**	**−2.00**	**0.045**
	**Emotional Lability**	**1.09**	**0.32**	**3.45**	**< 0.001**
	Detachment	0.28	0.35	0.80	0.423
	Age	−0.003	0.36	−0.01	0.994
	Sex (female)	0.20	0.56	0.36	0.716
	IQ	0.17	0.30	0.56	0.577

*Note:* Bold values indicate statistical significance at *p* < 0.05.

*PID‐5 three‐domain model*: Detachment was positively associated with NSSI.

*Three‐facet PID‐5 models*: Negative Affectivity facet Emotional Lability was positively associated with NSSI.

*Final models*: In the joint model, CATI Self‐Regulatory (Repetitive) Behaviors and PID‐5 Emotional Lability were positively associated with NSSI, whereas CATI Communication was inversely associated with NSSI. Effects were unchanged with covariates. The CATI Social Interactions association observed in the CATI‐only screen and the PID‐5 Detachment effect in the domain‐level model were both attenuated in the joint model, potentially due to suppression (i.e., shared variance between the scales).

#### 3.1.2 Lifetime Suicide Attempts (Table 3)

*CATI six‐subscale model*: Social Interactions was positively associated with lifetime attempts.

**TABLE 3:** Predictors of lifetime suicide attempts in 102 autistic adults: CATI screens, PID‐5 screens, and joint logistic models with standardized predictors.

Model	Predictor	Estimate	SE	*z*	*p*
CATI six‐subscale	Self‐Regulatory (Repetitive) Behaviors	−0.11	0.49	−0.23	0.817
	Cognitive Flexibility	−0.40	0.36	−1.11	0.269
	Social Camouflage	0.19	0.41	0.45	0.655
	Sensory Sensitivity	0.40	0.44	0.92	0.357
	**Social Interactions**	**0.84**	**0.37**	**2.26**	**0.023**
	Communication	0.31	0.37	0.82	0.410
PID‐5 domains	Disinhibition	−0.18	0.28	−0.66	0.512
	Detachment	0.38	0.24	1.56	0.118
	**Negative Affectivity**	**0.62**	**0.27**	**2.25**	**0.024**
PID‐5 facets	**Emotional Lability**	**0.59**	**0.29**	**2.00**	**0.046**
	Anxiousness	−0.34	0.32	−1.07	0.286
	Separation Insecurity	0.30	0.27	1.11	0.269
	Withdrawal	−0.05	0.32	−0.17	0.864
	**Anhedonia**	**1.05**	**0.33**	**3.21**	**0.001**
	Intimacy Avoidance	−0.16	0.25	−0.65	0.519
	Irresponsibility	−0.37	0.26	−1.39	0.164
	Impulsivity	0.18	0.29	0.61	0.544
	Distractibility	−0.45	0.33	−1.36	0.175
Final (unadjusted)	Social Interactions	0.67	0.38	1.78	0.075
	**Anhedonia**	**0.60**	**0.26**	**2.32**	**0.021**
	Emotional Lability	0.35	0.24	1.45	0.148
Final (adjusted for age, sex, IQ)	**Social Interactions**	**0.87**	**0.41**	**2.11**	**0.035**
	**Anhedonia**	**0.69**	**0.28**	**2.46**	**0.014**
	Emotional Lability	0.27	0.25	1.11	0.267
	Age	−0.34	0.30	−1.14	0.253
	Sex (female)	0.03	0.51	0.07	0.946
	**IQ**	**−0.57**	**0.26**	**−2.15**	**0.031**

*Note:* Bold values indicate statistical significance at *p* < 0.05.

*PID‐5 three‐domain model*: Negative Affectivity was positively associated with lifetime attempts.

*Three‐facet PID‐5 models*: Detachment facet, Anhedonia and Negative Affectivity facet Emotional Lability were each positively associated with attempts in facet screens.

*Final models*: In the joint model, PID‐5 Anhedonia was positively associated with lifetime attempts. With covariates, both PID‐5 Anhedonia and CATI Social Interactions were positively associated with lifetime attempts. In contrast, IQ was inversely associated with lifetime attempts.

#### 3.1.3 Lifetime Suicidal Ideation Intensity (Table 4)

*CATI six‐subscale model*: No subscale reached statistical significance.

**TABLE 4:** Predictors of worst‐episode suicidal ideation intensity in 102 autistic adults: CATI screens, PID‐5 screens, and joint linear models with standardized predictors.

Model	Predictor	Estimate	SE	*t*	*p*
CATI six‐subscale	Self‐Regulatory (Repetitive) Behaviors	0.62	1.07	0.58	0.565
	**Cognitive Flexibility**	**−1.53**	**0.73**	**−2.11**	**0.038**
	Social Camouflage	0.33	0.82	0.41	0.684
	Sensory Sensitivity	0.34	0.94	0.36	0.722
	**Social Interactions**	**1.67**	**0.71**	**2.34**	**0.022**
	Communication	−0.07	0.75	−0.09	0.928
PID‐5 domains	Disinhibition	−0.40	0.56	−0.71	0.479
	Detachment	0.97	0.50	1.95	0.055
	**Negative Affectivity**	**1.38**	**0.55**	**2.53**	**0.013**
PID‐5 facets	Emotional Lability	1.05	0.53	1.98	0.051
	Anxiousness	0.18	0.61	0.29	0.772
	Separation Insecurity	0.58	0.49	1.18	0.242
	Withdrawal	−0.86	0.56	−1.54	0.128
	**Anhedonia**	**1.56**	**0.56**	**2.80**	**0.006**
	Intimacy Avoidance	0.69	0.45	1.53	0.130
	Irresponsibility	−0.75	0.50	−1.50	0.136
	Impulsivity	−0.04	0.55	−0.08	0.935
	Distractibility	0.13	0.60	0.22	0.827
Final (unadjusted)	Cognitive Flexibility	−1.09	0.60	−1.81	0.073
	Negative Affectivity	0.92	0.50	1.82	0.073
	Social Interactions	1.03	0.63	1.64	0.105
	Anhedonia	0.88	0.51	1.73	0.087
Final (adjusted for age, sex, IQ)	Cognitive Flexibility	−1.01	0.62	−1.62	0.110
	Negative Affectivity	0.75	0.52	1.44	0.154
	**Social Interactions**	**1.43**	**0.69**	**2.07**	**0.042**
	Anhedonia	0.99	0.51	1.93	0.058
	Age	−0.52	0.52	−1.01	0.314
	Sex (female)	0.56	0.94	0.59	0.554
	IQ	−0.50	0.48	−1.03	0.306

*Note:* Bold values indicate statistical significance at *p* < 0.05.

*CATI six‐subscale model*: Cognitive Flexibility was negatively associated with ideation intensity, whereas Social Interactions was positively associated with ideation intensity.

*PID‐5 three‐domain model*: Negative Affectivity was positively associated with ideation intensity.

*Three‐facet PID‐5 models*: Detachment facet Anhedonia was positively associated with ideation intensity.

*Final models*: In the joint model without covariates, no predictor reached significance. With covariates, CATI Social Interactions was positively associated with ideation intensity, though the effect was weaker than in the CATI‐only model. Once again, this suggests potential suppression between predictors in the joint model (i.e., shared variance between the scales).

#### 3.1.4 Lifetime Suicidal Ideation Severity (Table 5)

*PID‐5 three‐domain model*: Detachment and Negative Affectivity were positively associated with ideation severity.

**TABLE 5:** Predictors of lifetime suicidal ideation severity in 102 autistic adults: CATI screens, PID‐5 screens, and joint linear models with standardized predictors.

Model	Predictor	Estimate	SE	*t*	*p*
CATI six‐subscale	Self‐Regulatory (Repetitive) Behaviors	0.00	0.35	0.01	0.991
	Cognitive Flexibility	−0.17	0.26	−0.63	0.530
	Social Camouflage	0.04	0.30	0.12	0.904
	Sensory Sensitivity	0.18	0.32	0.57	0.567
	Social Interactions	0.32	0.25	1.27	0.207
	Communication	0.27	0.28	0.97	0.334
PID‐5 domains	Disinhibition	−0.10	0.20	−0.52	0.604
	**Detachment**	**0.55**	**0.17**	**3.25**	**0.002**
	**Negative Affectivity**	**0.44**	**0.19**	**2.38**	**0.019**
PID‐5 facets	**Emotional Lability**	**0.52**	**0.17**	**2.99**	**0.004**
	Anxiousness	−0.15	0.18	−0.79	0.433
	Separation Insecurity	0.12	0.16	0.75	0.453
	Withdrawal	−0.34	0.19	−1.81	0.073
	**Anhedonia**	**0.79**	**0.18**	**4.32**	**< 0.001**
	Intimacy Avoidance	0.29	0.15	1.92	0.058
	Irresponsibility	−0.07	0.17	−0.39	0.694
	Impulsivity	0.03	0.18	0.15	0.879
	Distractibility	−0.25	0.20	−1.23	0.221
Final (unadjusted)	**Anhedonia**	**0.62**	**0.16**	**3.96**	**< 0.001**
	**Emotional Lability**	**0.42**	**0.16**	**2.68**	**0.009**
Final (adjusted for age, sex, IQ)	**Anhedonia**	**0.65**	**0.16**	**4.11**	**< 0.001**
	**Emotional Lability**	**0.46**	**0.16**	**2.94**	**0.004**
	Age	−0.10	0.17	−0.57	0.567
	Sex (female)	−0.20	0.31	−0.63	0.528
	IQ	0.04	0.16	0.26	0.792

*Note:* Bold values indicate statistical significance at *p* < 0.05.

*PID‐5 three‐facet models*: Detachment facet, Anhedonia and Negative Affectivity facet, Emotional Lability were positively associated with ideation severity.

*Final models*: In the joint model, both PID‐5 Anhedonia and PID‐5 Emotional Lability were positively associated with ideation severity. Both effects remained significant with covariates.

#### 3.1.5 Ideation‐to‐Attempt Transition (*n* = 79; Table 6)

*CATI six‐subscale model*: Social Interactions was significantly higher in people with a lifetime suicide attempt relative to lifetime suicidal ideation.

**TABLE 6:** Predictors of ideation‐to‐attempt transition among 79 participants with lifetime ideation: CATI screens, PID‐5 screens, and joint logistic models with standardized predictors.

Model	Predictor	Estimate	SE	*z*	*p*
CATI six‐subscale	Self‐Regulatory (Repetitive) Behaviors	0.17	0.61	0.27	0.787
	Cognitive Flexibility	−0.74	0.42	−1.77	0.077
	Social Camouflage	0.29	0.44	0.66	0.512
	Sensory Sensitivity	0.20	0.52	0.38	0.703
	**Social Interactions**	**0.91**	**0.41**	**2.21**	**0.027**
	Communication	0.04	0.42	0.09	0.926
PID‐5 domains	Disinhibition	0.05	0.31	0.16	0.877
	Detachment	0.33	0.28	1.21	0.228
	Negative Affectivity	0.49	0.31	1.60	0.109
PID‐5 facets	**Emotional Lability**	**0.67**	**0.33**	**2.08**	**0.041**
	Anxiousness	−0.35	0.36	−0.97	0.332
	Separation Insecurity	0.24	0.29	0.83	0.404
	Withdrawal	−0.03	0.34	−0.08	0.939
	**Anhedonia**	**1.01**	**0.35**	**2.90**	**0.004**
	Intimacy Avoidance	−0.12	0.27	−0.43	0.669
	Irresponsibility	−0.16	0.29	−0.54	0.591
	Impulsivity	0.04	0.32	0.12	0.903
	Distractibility	−0.16	0.35	−0.46	0.643
Final (unadjusted)	**Anhedonia**	**0.61**	**0.29**	**2.11**	**0.035**
	Emotional Lability	0.45	0.27	1.65	0.100
	Social Interactions	0.61	0.40	1.54	0.123
Final (adjusted for age, sex, IQ)	**Anhedonia**	**0.72**	**0.31**	**2.30**	**0.022**
	Emotional Lability	0.39	0.28	1.37	0.170
	Social Interactions	0.81	0.44	1.83	0.068
	Age	−0.27	0.33	−0.83	0.409
	Sex (female)	0.38	0.56	0.68	0.497
	**IQ**	**−0.55**	**0.28**	**−1.97**	**0.048**

*Note:* Bold values indicate statistical significance at *p* < 0.05.

*PID‐5 three‐domain model*: No domain reached statistical significance.

*PID‐5 three‐facet models*: Detachment facet, Anhedonia and Negative Affectivity facet, Emotional Lability, were each significantly higher in people with lifetime suicide attempts relative to lifetime suicidal ideation.

*Final models*: In the joint model, PID‐5 Anhedonia was higher among those with attempts than ideation only, though the effect was weaker than in the facet‐only model. Emotional Lability and CATI Social Interactions did not reach statistical significance in joint models. Higher IQ showed a marginal inverse association after covariate adjustment.

**FIGURE 1:**
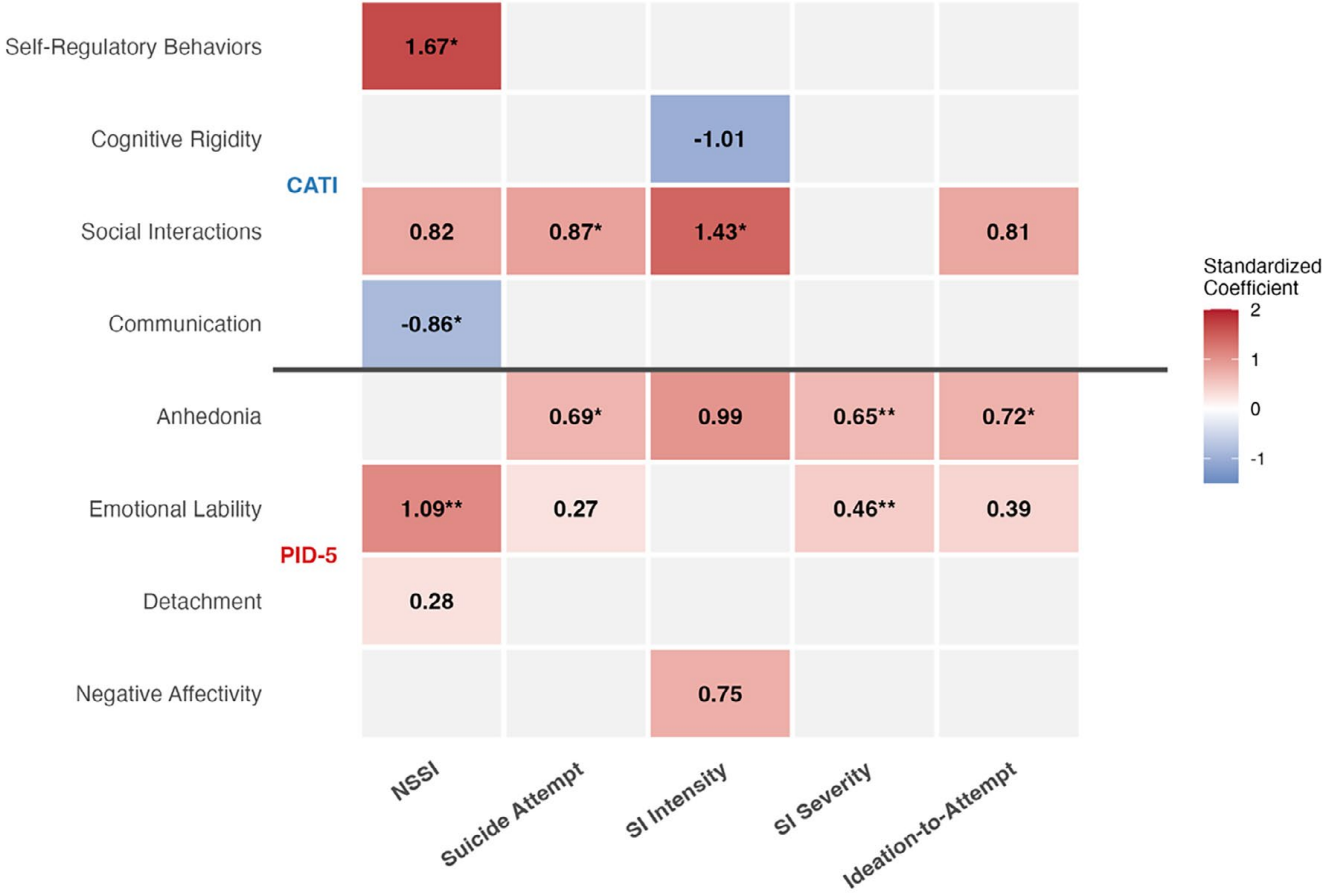
Trait‐outcome associations across self‐injurious thoughts and behaviors. *Note:* Cells display standardized regression coefficients from final models adjusted for age, sex, and IQ. Color intensity indicates effect magnitude: Red = positive associations (higher trait scores predict greater risk/severity); blue = negative associations; gray = predictor not included in that model. Significance levels: *p < 0.05, **p < 0.01. The horizontal line separates CATI subscales (top) from PID‐5‐SF facets and domains (bottom). Empty cells indicate predictors excluded from the final adjusted model by stepwise selection. CATI = Comprehensive Autistic Trait Inventory; NSSI = nonsuicidal self‐injury; PID‐5‐SF = Personality Inventory for DSM‐5 Short Form; SI = suicidal ideation.

Figure 1 displays the standardized regression coefficients from the final adjusted models for all trait predictors across the five SITB outcomes, illustrating the pattern of associations and highlighting both shared and outcome‐specific predictors.

#### 3.1.6 Exploratory Analyses (Latent Factors)

Exploratory latent‐variable models fit in Mplus (Muthén and Muthén 2019) tested whether shared variance between CATI Social Interactions and PID‐5 Detachment/Anhedonia contributed to the suppression we observed in joint regressions. We first fit measurement models using Bayesian confirmatory factor analysis. In the first model, we specified a latent Detachment factor from the PID‐5 Detachment facets (Withdrawal, Intimacy Avoidance, Anhedonia) and the CATI Social Interactions. The model was an excellent fit to the data (CFI = 1.00, RMSEA < 0.001), with all scales loading significantly on the latent Detachment factor. In the second model, we specified a latent Anhedonia factor from individual PID‐5 Anhedonia items and the CATI Social Interactions scale. The second model also demonstrated excellent fit to the data (CFI = 1.00, RMSEA < 0.001), with all indicators having significant factor loadings. Next, we substituted the latent Detachment and Anhedonia factors into the models where we had observed suppression in place of CATI Social Interactions and PID‐5 Detachment or Anhedonia, respectively (all covariates were retained). Latent Detachment significantly predicted lifetime NSSI (*β* = 0.46, *p* = 0.036, 95% CI = 0.06, 0.74). Likewise, latent Anhedonia significantly predicted ideation intensity (*β* = 0.29, *p* < 0.001, 95% CI = 0.07, 0.50) and was higher among those with attempts than ideation only (*β* = 0.44, *p* < 0.001, 95% CI = 0.17, 0.66). This suggests that CATI Social Interactions shares variance with both the higher‐order Detachment domain and the lower‐order Anhedonia facet; whereas the former predicts NSSI, the latter predicts ideation intensity and attempt history (relative to ideation only). Moreover, shared variance between these scales accounts for their attenuated effects in the joint models reported above.

## 4 Discussion

This study integrated an autism‐informed trait inventory with a broader trait taxonomy‐informed measure to identify personality correlates of SITB in autistic adults. Three patterns were consistent across models. First, Detachment facet Anhedonia was associated with higher ideation severity and higher odds of lifetime suicide attempts and the ideation‐to‐attempt transition. Second, the Negative Affectivity facet Emotional Lability was a strong correlate of NSSI and ideation severity. Third, several CATI domains added complementary information, with higher Social Interactions scores predicting greater risk across most outcomes, Self‐Regulatory (Repetitive) Behaviors scores predicting NSSI, and Communication scores inversely relating to NSSI. After covariate adjustment, higher IQ also showed a small protective association with lifetime suicide attempts. Notably, exploratory latent‐factor models demonstrated that shared variance between PID‐5 Detachment/Anhedonia and CATI Social Interactions was predictive of several outcomes. Thus, while both autism‐specific traits and broader personality dimensions provide incremental validity for SITB in autism, there is also important overlap between the two, highlighting a partially shared underlying trait architecture.

Anhedonia was the most consistent correlate of risk in the joint models. Overall, the Anhedonia facet reflects low interest and enjoyment in routine activities (e.g., “Nothing seems to interest me very much,” “I almost never enjoy life”). Converging work in non‐autistic samples links anhedonia and broader reward‐related deficits to persistent suicidal ideation and attempts, with effects seen across questionnaires and experimental paradigms that probe reward responsivity, learning, and valuation (e.g., Antezana et al. 2024; Bettis et al. 2022; Ducasse et al. 2018; Tsypes et al. 2018, 2019, 2021). Reward blunting present in anhedonia may reduce the perceived value of alternatives to suicide, weaken deterrents (reasons for living), and sustain suicidal thinking. Future work is needed to better understand the contributors to self‐reported anhedonia in autistic adults.

Emotional lability showed a robust association with NSSI. This facet indexes ease of becoming emotionally escalated, along with rapid and unpredictable shifts in affect (e.g., “I get emotional easily, often for very little reason,” “My emotions are unpredictable”), which is distinct from sustained negative mood or anxiety. This NSSI link is in line with functional models that view self‐injury as a negatively reinforced behavior that provides relief from acute aversive arousal, supported by empirical work that shows affect regulation as a primary function of NSSI (Hamza and Willoughby 2015; Kuehn et al. 2022). Meta‐analytic and longitudinal studies in non‐autistic samples connect NSSI to high negative affect and to difficulties modulating fast affective shifts, with additional contributions from impulsivity in some cohorts (Hamza et al. 2015; Hamza and Willoughby 2015; Kuehn et al. 2022; You et al. 2016). Autism research describes pervasive dysregulation in contexts of sensory overload, social effort, and masking, which may plausibly shorten the time from trigger to peak arousal (Beck et al. 2024; Lai 2023). Taken together, the present findings indicate that rapidly rising affect is a proximal target when NSSI is present in autistic adults. Accordingly, clinicians should not infer benignity from autism status or from whether a behavior looks “autism‐related.” Instead, self‐injury in autistic adults should prompt direct assessment of intent, function, and suicide risk.

Similar to Anhedonia, CATI Social Interactions was associated with a higher risk across SITB. Higher scores on this scale reflect greater difficulty enjoying and seeking contact, greater avoidance, more stress in social situations, and lower confidence in making or keeping friends (e.g., “I generally enjoy social events” [reverse‐scored], “I find social interactions stressful”). This pattern is consistent with contemporary theories that highlight the centrality of interpersonal processes in risk for SITB (Klonsky and May 2015; O'Connor and Kirtley 2018; Van Orden et al. 2010) and with qualitative accounts of sustained social effort, misunderstanding, and invalidation in autistic adults that can deplete coping resources (Beck et al. 2024; Cassidy et al. 2018). For NSSI, CATI Self‐Regulatory (Repetitive) Behaviors, which index stress‐triggered rocking, pacing, fiddling, nail biting, and hair pulling, was positively related to NSSI, consistent with a shared negative‐reinforcement function. Frequent reliance on repetitive motor acts to down‐regulate arousal may co‐occur with NSSI and, for some people, may involve a transition from these repetitive acts to NSSI that serves the same regulatory goal. Future work should delineate functional similarities and differences between stimming and NSSI in autistic adults (Antezana et al. 2025). In contrast, after accounting for variance shared with CATI Social Interactions and Emotional Lability, the residual Communication signal emerged as protective against NSSI engagement, and future work will be needed to replicate and better understand this effect.

Using CATI alongside PID‐5 achieved complementary coverage. CATI quantified domains central to autistic experience that are not fully represented by broad trait models, including masking, sensory sensitivity, cognitive flexibility, and repetitive regulation (English et al. 2021, 2025). Conversely, PID‐5 provided facet‐level resolution of affective and regulatory tendencies that are well studied transdiagnostically, but often not in autism specifically (Krueger et al. 2012). Prior work suggests only partial alignment between autistic traits and broad personality dimensions, with social autistic traits mapping most closely to Extraversion/Detachment and other features less well captured by the Big Five and related spectra (Lodi‐Smith et al. 2019; Michelini et al. 2024; Stanton et al. 2021; Widjaja et al. 2025). The present findings are consistent with that pattern, such that autism‐specific traits added unique information beyond PID‐5, while facet‐level PID‐5 facets Anhedonia and Emotional Lability emerged as important correlates of risk for SITB in combined models. There was also evidence for partial overlap between the measures. Our exploratory latent‐factor models revealed a close mapping between PID‐5 Detachment/Anhedonia and CATI Social Interactions. When these scales were entered together in joint models, their predictive utility was frequently attenuated, sometimes to the point of becoming null. This pattern is consistent with statistical suppression and suggests that the variance shared by these constructs carries the predictive signal that is obscured when they are entered separately in joint models. Indeed, a latent Detachment factor that included loadings from all Detachment facets and CATI Social Interactions significantly predicted lifetime NSSI, whereas a latent Anhedonia factor spanning all Anhedonia items and CATI Social Interactions predicted ideation intensity and attempt history (relative to ideation only). Overall, our findings highlight the utility of incorporating features unique to autism into broadband dimensional models, while also identifying points of convergence between these measurement systems, particularly with regard to reward responsiveness and sociability.

Disinhibition in the PID‐5 is reflective of problems with planful behavior, a tendency to act without forethought, and distractibility. Contrary to our hypotheses, neither this domain nor its facets were related to any aspects of SITB. Context is important when interpreting these findings. First, the literature on impulsivity and SITB is often mixed, with effects varying by measure and construct definition. Relatedly, there is also significant heterogeneity in executive functioning within autistic adults (e.g., Geurts et al. 2014). Second, autistic trait configuration may shift the ideation‐to‐attempt trajectory. For example, preference for sameness and difficulties with cognitive flexibility can oppose rash actions, making a transition from considering to attempting suicide more dependent on low reward valuation/responsiveness and interpersonal difficulties than on a tendency to act impulsively per se. Given these possibilities and pending replications, cautious interpretation rather than a conclusion that disinhibition is irrelevant for SITB in autistic adults is recommended.

The lack of robust sex or gender differences in our models aligns with the emerging consensus that, within autism, sex and gender are not reliable stratifiers of SITB risk in the way they often are in non‐autistic populations. Across large cohorts, suicide mortality is elevated for autistic people, and absolute suicide rates appear broadly comparable for autistic males and females (Hirvikoski et al. 2016; Kõlves et al. 2021; Kirby et al. 2024). For nonfatal outcomes, patterns of sex differences are more outcome‐dependent and heterogeneous across studies, with some cohorts showing higher attempt or self‐harm rates in autistic females and others showing weak or null differences (Kõlves et al. 2021; Brown et al. 2024). Meta‐analytic evidence further suggests that sample composition can moderate specific outcomes such as suicide plans and suicidal ideation, rather than sex operating as a consistent moderator across all SITB outcomes (Newell et al. 2023). Youth‐focused syntheses similarly conclude that age and sex do not explain autistic youths' vulnerability to suicidality (O'Halloran et al. 2022). Together, the literature supports the interpretation that sex and gender effects in autism are outcome‐ and ascertainment‐dependent and may be smaller than commonly assumed, or obscured by, heterogeneity linked to measurement, clinical enrichment, co‐occurring psychopathology, and interpersonal‐contextual risk and protective factors (Mournet et al. 2023; Brown et al. 2024).

Several limitations that inform future research directions should be acknowledged. The data were cross‐sectional and lifetime in scope, which limits causal inference and may introduce recall bias. Although we discuss the ideation‐to‐action transition in one set of analyses, that comparison relies on lifetime histories of ideation and attempts and therefore does not establish temporal ordering or within‐person transition dynamics. Suicidal ideation also fluctuates over time (e.g., Coppersmith et al. 2023; Hallensleben et al. 2019; Kaurin et al. 2023; Kleiman et al. 2017), which warrants future ecological momentary assessment work to probe how personality traits influence dynamic patterns of suicidal thinking in daily life. In addition, although personality traits are relatively stable, they do not exist in a vacuum and likely interact with life experiences and environmental factors that the present study did not capture. Further, anhedonia and emotional lability are mechanistically complex constructs that reflect the influence of multiple underlying processes, which should be examined in future research. Finally, the sample was predominantly female and had relatively high IQ, and the generalizability of our results to other samples should be investigated.

Despite these limitations, the study had several notable strengths with direct implications for research and practice. First, it included a large, clinician‐verified autistic sample confirmed by ADOS‐2. Second, the high‐risk nature of this sample allowed a direct comparison between people with ideation only and those with attempts, which is a rare contrast in autism suicide research. Third, the stepwise analytic design strategy is novel and shows the merit of combining autistic and general personality traits. This integrated frame moves the field from prevalence toward a trait‐informed perspective on understanding risk for SITB in autistic adults. Clinically, the work supports adding brief screening for anhedonia and emotional lability to routine assessments, using autism‐adapted behavioral activation to address low reinforcement and withdrawal (Bal et al. 2025), and pairing rapid arousal‐reduction skills with brief, individualized crisis plans (e.g., autism‐adapted safety plans or emotional support plans) to manage acute affective surges (Bal et al. 2024; Rodgers et al. 2024). When dysregulation and self‐injury are persistent, referral to structured emotion regulation interventions such as Dialectical Behavior Therapy (DBT) skills training (Linehan 2015; Ritschel et al. 2022) or the Emotion Awareness and Skills Enhancement (EASE) Program (White et al. 2025) may be indicated.

## Funding

This work was supported by the NIH (P50MH130957, K23MH130664, K01MH123915, T32MH018951, UL1TR001857).

## Ethics Statement

All participants provided written informed consent prior to study participation, and the Institutional Review Board approved all procedures.

## Conflicts of Interest

The authors declare no conflicts of interest.

## Data Availability

Data are available in the NIMH Data Archive NDA Collection C4550.

## Endnotes

^1^
We recognize that some DSM and PID‐5 domain and facet labels may not align with neuro‐affirming perspectives in autism (Bottema‐Beutel et al. 2021). We retain these terms only to refer to the validated scales we administered and not to define participants. Throughout, we aim to minimize deficit framing and follow community guidance to explain our language choices and to respect the preferences of autistic people, many of whom favor identity‐first terminology (Bottema‐Beutel et al. 2021). Future work should continue to refine terminology in partnership with autistic communities.

^2^
When gender was substituted for sex, the overall pattern of results remained unchanged.
